# Inhibitory Effects of Short-Chain Fatty Acids and ω-3 Polyunsaturated Fatty Acids on Profibrotic Factors in Dermal Fibroblasts

**Published:** 2019-03-01

**Authors:** Noriaki Maeshige, Kazuhiro Torii, Hiroto Tabuchi, Midori Imai, Yuka Koga, Mikiko Uemura, Michiko Aoyama-Ishikawa, Makoto Miyoshi, Hidemi Fujino, Hiroto Terashi, Makoto Usami

**Affiliations:** ^a^Department of Rehabilitation Science; ^b^Division of Nutrition and Metabolism, Department of Biophysics, Kobe University Graduate School of Health Sciences, Kobe, Japan; ^c^Department of Plastic Surgery, Kobe University Graduate School of Medicine, Kobe, Japan; ^d^Faculty of Clinical Nutrition and Dietetics, Konan Women’s University, Kobe, Japan

**Keywords:** ω-3 polyunsaturated fatty acid, short-chain fatty acid, dermal fibroblast, pro-fibrotic factor, proinflammatory factor

## Abstract

**Objective:** Dermal fibroproliferative disorders impair patients’ quality of life. Although several therapeutic approaches exist for treatment of dermal scars, the development of effective ointments with few adverse effects could improve these therapeutic methods. Short-chain and ω-3 polyunsaturated fatty acids are reported to be immunomodulators with anti-inflammatory properties. Our aim was to evaluate anti-inflammatory and antifibrogenic effects of these fatty acids in human dermal fibroblasts. **Methods:** Cells were incubated with short-chain fatty acids (butyrate or propionate; 0-16 mM) and/or ω-3 polyunsaturated fatty acids (docosahexaenoic acid or eicosapentaenoic acid; 0-100 μM) for 24 hours to evaluate antifibrogenic effects and for 3 or 48 hours to evaluate anti-inflammatory effects after stimulation with lipopolysaccharide or without stimulation. Expression levels of α-smooth muscle actin, collagen I, collagen III, and IL-6 were evaluated, as were cell proliferation, stress fiber formation, and histone acetylation. **Results:** In the lipopolysaccharide-unstimulated group, butyrate inhibited mRNA expression of α-smooth muscle actin and collagen III more effectively than propionate and increased histone acetylation. Docosahexaenoic acid inhibited mRNA expression of α-smooth muscle actin and collagen III, whereas eicosapentaenoic acid did not. Combining butyrate with docosahexaenoic acid had stronger effects, downregulating α-smooth muscle actin, collagen I, and collagen III mRNA. As for cell proliferation and stress fiber formation, butyrate acted as a stronger inhibitor than docosahexaenoic acid and the combined administration had stronger effects. In the lipopolysaccharide-stimulated group, butyrate and docosahexaenoic acid attenuated IL-6 mRNA upregulation by lipopolysaccharide. **Conclusion:** Butyrate and docosahexaenoic acid may be a novel therapeutic approach to treatment of dermal fibroproliferative disorders.

Dermal fibroproliferative disorders, such as hypertrophic scars, impair the patients’ quality of life owing to cosmetic and functional deficiencies.^1^ These conditions are caused by aberrant wound healing after any injury or skin incision and are characterized by hyperproliferation of dermal fibroblasts and overproduction of the extracellular matrix, especially type I collagen (collagen I) and collagen III.^2^ α-Smooth muscle actin (α-SMA) expression and stress fiber formation are observed in the cytoplasm of activated fibroblasts, which undergo intense fibrogenesis, and transforming growth factor β1 (TGF-β1) is reported to be one of the augmenting factors of dermal fibrosis.^3^ Therefore, regulation of these factors is important for preventing the progression of fibrosis. Furthermore, chronic inflammation activates the aforementioned fibrogenic responses.^4^ In particular, overexpression of the *IL-6* gene in hypertrophic scar fibroblasts has been reported.^5^ Therefore, regulation of inflammatory responses is needed for scar management. Several therapeutic modalities exist for preventing hypertrophic scar formation, including silicon-based products and radiation therapy, and multiple therapeutic approaches are applied in response to a patient's symptoms.^6^ Although intraregional treatments using steroids or 5-fluorouracil are known to have promising therapeutic effects,^7^ the adverse effects of these treatments are considered potentially problematic.

Short-chain fatty acids (SCFAs) are the end products of anaerobic bacterial fermentation of indigestible carbohydrates in the colon.^8^ Predominantly, butyrate and propionate possess strong physiological activities as histone deacetylase (HDAC) inhibitors.^8^ We have revealed the inhibitory effects of butyrate and propionate on nuclear factor kappa B (NF-κB) activation in peripheral blood mononuclear cells.^9^ Recently, the antifibrogenic effect of butyrate in several mesenchymal, rat pancreatic, or hepatic stellate cells was reported,^10^^,^^11^ revealing inhibition of cell growth, collagen III production, and α-SMA expression. However, SCFAs effective at suppression of dermal fibrogenesis are unknown.

Docosahexaenoic acid (DHA) and eicosapentaenoic acid (EPA) are primary fatty acids among ω-3 polyunsaturated fatty acids (PUFAs) found in products derived from marine organisms, including fish oil. Higher levels of arachidonic acid, one of the ω-6 PUFAs, have been detected in hypertrophic scars compared with healthy dermis samples.^12^ Although ω-6 PUFAs possess proinflammatory effects,^13^ ω-3 PUFAs have anti-inflammatory effects via their lipid mediators.^14^ Therefore, these fatty acids could be an effective treatment option for dermal fibroproliferative disorders. The antifibrogenic effect of DHA in human peritoneal fibroblasts has been reported, including inhibition of vascular endothelial growth factor and collagen I expression.^15^ Although both DHA and EPA are purported to possess individual therapeutic activities, the effectiveness of these fatty acids against dermal fibrosis remains unclear.

We hypothesized that SCFAs and ω-3 PUFAs possess inhibitory effects on the expression of profibrotic and proinflammatory factors in dermal fibroblasts. In this study, our objective was to investigate the possible antifibrogenic and anti-inflammatory effects of SCFAs (butyrate and propionate) and ω-3 PUFAs (DHA and EPA) and of their combined administration in human dermal fibroblasts (HDFs). To evaluate the anti-inflammatory effects, we stimulated HDFs with lipopolysaccharide (LPS; derived from *Pseudomonas aeruginosa*), which is the major pathogenic factor of burn infections.^16^

## MATERIALS AND METHODS

### Cell culture

HDFs (CC-2511; Clonetics, San Diego, Calif) were grown at 37°C and 5% CO_2_ in 100-mm tissue culture dishes (Iwaki, Tokyo, Japan) containing Dulbecco's modified Eagle's medium (DMEM; Dainippon Sumitomo Pharma, Osaka, Japan) supplemented with 10% of fetal bovine serum (FBS) (Nichirei, Tokyo, Japan), penicillin (50 U/mL), and streptomycin (50 μg/mL; MP Biomedicals, Illkirch, France). The cells at passages 5 to 8 were used for experimentation. Because normal fibroblasts increase own α-SMA expression, stress fiber formation, and proliferation when cultured on a plastic substrate in the presence of FBS,^3^ we seeded HDFs in plastic plates with 10% of FBS to evaluate the suppressive effects of SCFAs and PUFAs on these factors. The cell concentrations were 2.8 × 10^5^/well in 6-well flat-bottom plates (Iwaki) and 8.0 × 10^3^/well in 96-well flat-bottom plates (Iwaki). After 24 hours, fatty acids were added and cells were incubated for additional 24 hours to evaluate the antifibrogenic effects because of the absence of inhibitory effects at 12 hours on mRNA expression of profibrotic factors in our preliminary experiments. Anti-inflammatory effects were evaluated at 3 to 48 hours after administration of LPS and fatty acids. HDFs were subsequently processed for total RNA isolation using the TRIzol Reagent (Invitrogen, Carlsbad, Calif) and for protein isolation using ProPrep (iNtRON, Gyeonggi-do, South Korea). Trypan blue staining was performed to distinguish live cells from dead cells in order to calculate cell viability.

### Fatty acid administration

Sodium butyrate (Sigma-Aldrich, St Louis, Mo) and sodium propionate (Sigma-Aldrich) served as SCFAs, whereas sodium DHA (Sigma-Aldrich) and sodium EPA (Sigma-Aldrich) were used as ω-3 PUFAs. To evaluate single-agent administration, butyrate or propionate was applied at a concentration of 0, 1, 4, or 16 mM and DHA or EPA was applied at a concentration of 0, 50, or 100 μM, based on our previous in vitro studies.^9^^,^^17^^-^21 To evaluate combined agent administration, butyrate at a concentration of 0, 1, 4, or 16 mM was applied in combination with DHA at a concentration of 100 μM.

### Proliferation assays

HDFs were seeded in 96-well plates and treated with fatty acids for 24 hours, followed by analysis using the 5-bromo-20-deoxyuridine (BrdU) incorporation assay (Roche, Basel, Switzerland).

### Quantitative real-time polymerase chain reaction analysis

RNA concentration and purity were determined by means of absorbance at 260 and 280 nm. First-strand cDNAs were synthesized from the isolated RNAs with the First-Strand cDNA Synthesis System for reverse transcription-polymerase chain reaction (PCR) (Invitrogen). cDNAs were used for subsequent quantitative real-time PCR analysis using the SYBR Premix Ex Taq II (Takara Bio, Otsu, Japan) with appropriate primers (Table 1). The PCR reactions were run on an iCycler IQ (Bio-Rad, Hercules, Calif) for 40 cycles at 95°C for 30 seconds, at an annealing temperature (Table 1) for 30 seconds, and at 72°C for 30 seconds. Post-PCR melting curves were confirmed by the specificity of single-target amplification. All measurements were done in duplicate. All specific quantities were normalized to *GAPDH*. For each sample, the threshold cycle (*C*_t_) was calculated on the basis of the cycle at which the fluorescence increased above a threshold level. The *C*_t_ values were calculated for every sample for the target genes as follows: *C*_t_ (target gene) − *C*_t_ (internal control gene); *GAPDH* served as the internal control gene. The relative expression level for 1 target gene (ΔΔ*C*_t_) was calculated by subtraction of the Δ*C*_t_ of each control sample from the Δ*C*_t_ of each experimental sample. Finally, the relative expression value, normalized to an endogenous reference, was calculated as 2^−ΔΔ^*^C^*^t^_._

### Western blotting

A solution of 5 μL of the extracted protein was used to measure protein concentration by Lowry's method (RC DC Protein Assay Kit; Bio-Rad). Western blotting was carried out to determine histone acetylation. In brief, samples containing equal amounts of protein were subjected to electrophoresis in 12% polyacrylamide gels, transferred to polyvinylidene difluoride membranes (GE Healthcare, Buckinghamshire, England), and blocked with 5% non-fat milk. The membranes were incubated with primary antibodies against acetylated histone (1:1000; Cell Signaling Technology Inc, Danvers, Colo) and GAPDH (1:40,000; Sigma-Aldrich) overnight at 4°C, followed by incubation with the appropriate horseradish peroxidase–conjugated secondary antibody for 1 hour at room temperature. The blots were then developed using the ECL-plus Western Blotting Detection System (GE Healthcare) and exposed on Hyperfilm (GE Healthcare). Densitometric results were analyzed using the ImageJ software (National Institutes of Health, Bethesda, Md).

### Immunofluorescent staining

Stress fibers and nuclear morphology in HDFs were analyzed by immunofluorescent staining. In this experiment, HDFs were seeded on culture cover slips (25-mm diameter; Matsunami Glass Inc, Ltd, Osaka, Japan). HDFs were treated with butyrate at a concentration of 0 or 16 mM in the presence or absence of 100 μM DHA and next were fixed in 4% paraformaldehyde for 30 minutes, followed by immersion in a blocking solution consisting of 1% FBS in Tris-buffered saline (TBS) for 10 minutes at room temperature. After a wash in TBS, F-actin was labeled with phallotoxins (1:40; Invitrogen) for 20 minutes. Finally, nuclei were stained with 4′,6-diamidino-2-phenylindole (DAPI; 1:1000 dilution; Dojin, Kumamoto, Japan). Immunofluorescent staining patterns were examined under a BX50 fluorescence microscope at ×200 magnification (Olympus, Tokyo, Japan) and recorded with a digital camera (EOS Kiss X4; Canon, Tokyo, Japan).

### Statistical analysis

The data are expressed as mean ± SEM. Differences were considered statistically significant at *P* < .05, as determined by the Tukey-Kramer post hoc test.

## RESULTS

### Stronger inhibition of α-SMA and collagen III mRNA expression by butyrate than by propionate in HDFs

**Figure 1:**
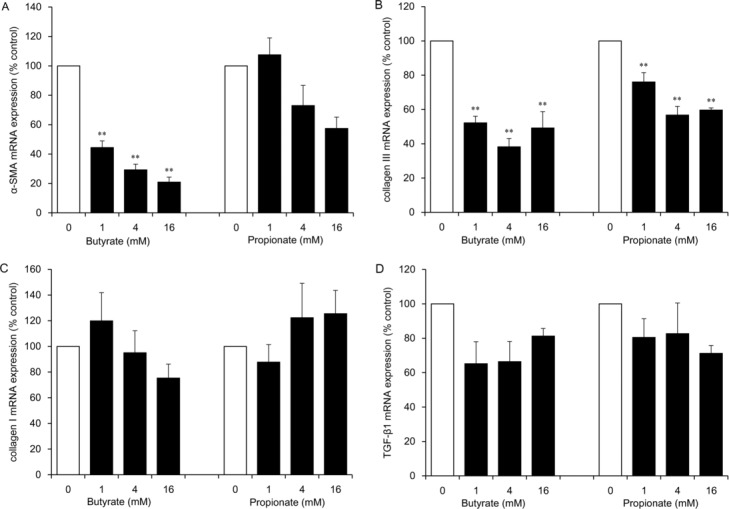
The effect of SCFAs (butyrate and propionate) on mRNA expression of α-SMA (*a*), collagen III (*b*), collagen I (*c*), and TGF-β1 (*d*) in HDFs. HDFs were exposed for 24 hours to each SCFA at the indicated concentrations. Data from 3 independent experiments were used to calculate mean values and SEMs. **P < .01 as compared with control cultures (Tukey-Kramer post hoc test). SCFA indicates short-chain fatty acid; α-SMA, α-smooth muscle actin; TGF-β1, transforming growth factor β1; and HDF, human dermal fibroblast.

Butyrate at concentrations of 1, 4, and 16 mM inhibited α-SMA mRNA expression in a significant and dose-dependent manner (to 44%, 29%, and 21% of the control level, respectively; *P* < .01) and collagen III mRNA expression (to 52%, 38%, and 49% of the control level, respectively; *P* < .01; Figs 1*a* and 1*b*). Propionate at concentrations of 11, 4, and 16 mM significantly inhibited collagen III mRNA expression (to 76%, 57%, and 60% of the control level, respectively; *P* < .01), which is a lower degree of inhibition than that observed with butyrate (Fig 1*b*). Significant differences in collagen I and TGF-β1 mRNA expression levels were not observed (Figs 1*c* and 1*d*).

### Inhibitory effects of DHA on α-SMA, TGF-β1, and collagen III mRNA expression levels in HDFs

**Figure 2:**
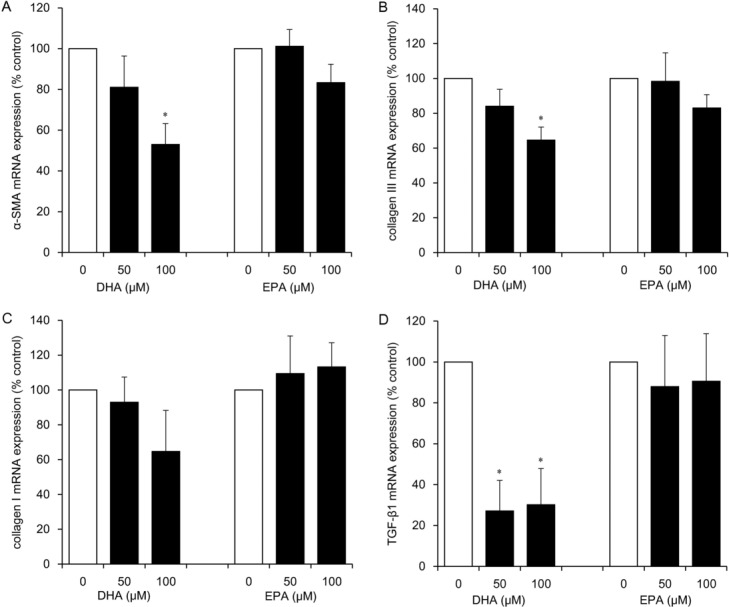
The effects of ω-3 PUFAs (DHA and EPA) on mRNA expression of α-SMA (*a*), collagen III (*b*), collagen I (*c*), and TGF-β1 (*d*) in HDFs. HDFs were exposed for 24 hours to each ω-3 PUFA at the indicated concentrations. Data from 3 independent experiments were used to calculate mean values and SEMs. *P < .05 as compared with control cultures (Tukey-Kramer post hoc test). ω-3 PUFA indicates ω-3 polyunsaturated fatty acid; DHA, docosahexaenoic acid; EPA, eicosapentaenoic acid; α-SMA, α-smooth muscle actin; TGF-β1, transforming growth factor β1; and HDF, human dermal fibroblast.

DHA at concentrations of 50 and 100 μM significantly inhibited mRNA expression of TGF-β1 (to 27% and 30% of the control level, respectively; *P* < .05) and at a concentration of 100 μM downregulated α-SMA (to 53% of the control level; *P* < .05) and collagen III (to 65% of the control level; *P* < .05; Figs 2*a* and 2*b*–2*d*). The inhibitory effects of 100 μM DHA on collagen I mRNA expression were not significant (Fig 2*c*), and no inhibitory effects of EPA were observed (Figs 2*a*–2*d*).

### Combined administration of butyrate and DHA exerts strong antifibrogenic effects in HDFs

**Figure 3:**
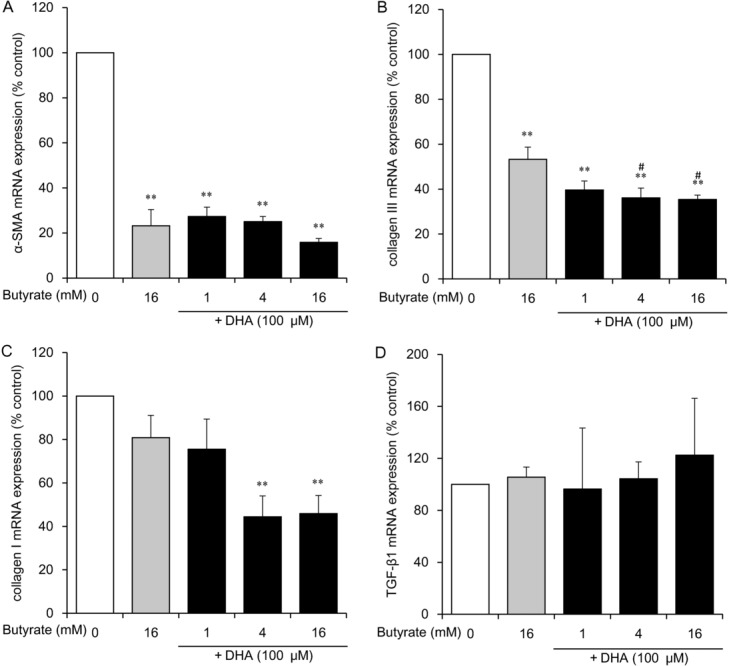
The effect of butyrate combined with DHA on mRNA expression of α-SMA (*a*), collagen III (*b*), collagen I (*c*), and TGF-β1 (*d*) in HDFs. HDFs were exposed for 24 hours to the indicated concentrations of butyrate with 100 μM DHA or to 16 mM butyrate without DHA. Data from 3 independent experiments were used to calculate mean values and SEMs. *P < .05, **P < .01 as compared with control cultures; *^#^P* < .01 in comparison with butyrate alone (Tukey-Kramer post hoc test). DHA indicates docosahexaenoic acid; α-SMA, α-smooth muscle actin; TGF-β1, transforming growth factor β1; and HDF, human dermal fibroblast.

We expected strong inhibitory effects of the combined administration of butyrate (1-16 mM) and DHA (100 μM). Butyrate at concentrations of 1, 4, and 16 mM combined with 100 μM DHA strongly inhibited mRNA expression of α-SMA (to 27%, 25%, and 16% of the control level, respectively; *P* < .01), collagen III (to 40%, 36%, and 35% of the control level, respectively; *P* < .01), and collagen I at concentrations of 4 and 16 mM (to 53% and 46% of the control level, respectively; *P* < .05; Figs 3*a*–3*c*). On the contrary, the combination of butyrate and DHA did not alter mRNA expression of TGF-β1 (Fig 3*d*).

**Figure 4:**
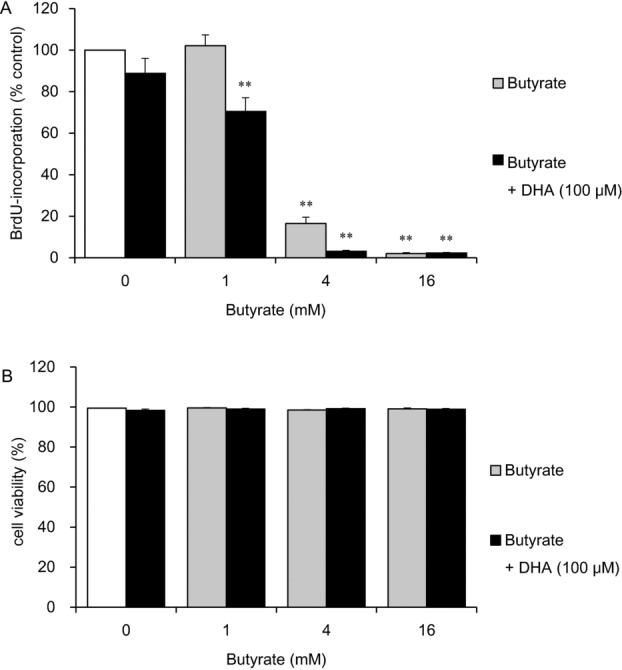
The effects of butyrate in the presence or absence of DHA on HDF cell proliferation and viability. HDFs were exposed for 24 hours to the indicated concentrations of butyrate in the presence or absence of 100 μM DHA. The cells were then labeled with BrdU for 24 hours, and proliferation was assessed by a BrdU DNA incorporation assay. Data from 8 cultures were used to calculate mean values and SEMs. **P < .01 as compared with control cultures (Tukey-Kramer post hoc test) (*a*). Cell viability was determined by trypan blue staining after fatty acid incubation for 24 hours. Data from 3 cultures were used to calculate mean values and SEMs (*b*). DHA indicates docosahexaenoic acid; HDF, human dermal fibroblast; and BrdU, 5-bromo-20-deoxyuridine.

Next, we assessed the effects of butyrate and DHA coadministration on cell proliferation (Fig 4*a*). Butyrate at concentrations of 4 and 16 mM significantly inhibited cell proliferation (to 17% and 2% of the control level, respectively; *P* < .01). Butyrate at a concentration of 1, 4, or 16 mM combined with 100 μM DHA significantly inhibited cell proliferation even more than did butyrate treatment alone (to 70%, 3%, and 2% of the control level, respectively; *P* < .01). Cell counting after trypan blue staining revealed that butyrate and DHA did not decrease cell viability, which remained more than 98% (Fig 4*b*).

### Histone acetylation after butyrate treatment of HDFs

**Figure 5:**
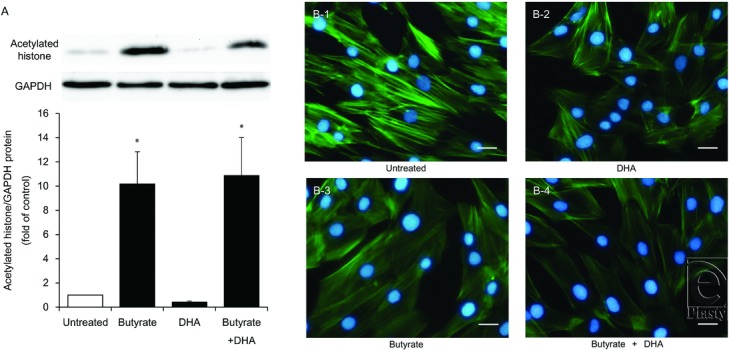
Histone acetylation under the influence of butyrate and disruption of stress fibers by butyrate and DHA in HDFs. HDFs were exposed to DHA (100 μM), butyrate (16 mM), or butyrate with DHA (16 mM and 100 μM, respectively). Acetylated histone protein was analyzed by Western blotting. A representative Western blot is shown at the top of each bar for each group and for the internal control. The graph presents the acetylated histone/GAPDH ratio. Data from 4 independent experiments were used to calculate mean values and SEMs. *P < .05 as compared with control cultures (Tukey-Kramer post hoc test) (*a*). Structural organization of F-actin and nuclei in HDFs was analyzed by immunofluorescent staining with phallotoxins (F-actin, green) and DAPI (nuclei, blue). The displayed micrographs, obtained by means of an Olympus BX50 fluorescence microscope at 200× magnification, are representative of all the cell cultures analyzed. Scale bar, 50 μm (*b*). DHA indicates docosahexaenoic acid; HDF, human dermal fibroblast; and DAPI, 4′,6-diamidino-2-phenylindole.

Butyrate at a concentration of 16 mM significantly increased histone acetylation by 10.2-fold (*P* < .05), but DHA did not. Combined administration of butyrate and DHA increased histone acetylation by 10.9-fold (*P* < .05) just as administration of butyrate alone did (Fig 5*a*).

### Stress fiber alteration by butyrate and DHA treatment of HDFs

Immunofluorescent staining was carried out to determine the localization of stress fibers (Fig 5*b*). Double staining for DNA (with DAPI) and F-actin indicated that 16 mM butyrate and 100 μM DHA each decreased stress fiber formation in HDFs, with the effect of butyrate being stronger. Furthermore, butyrate combined with DHA strongly disrupted stress fiber formation in the cytoplasm. To elucidate the effects of these fatty acids on apoptosis, we also evaluated the degree of DNA condensation and fragmentation in HDF nuclei. No increase in DNA condensation or fragmentation was observed after the combined butyrate + DHA treatment for 24 or 48 hours.

### Inhibition of IL-6 and profibrotic factor expression by butyrate and DHA and histone acetylation by butyrate in LPS-stimulated HDFs

**Figure 6:**
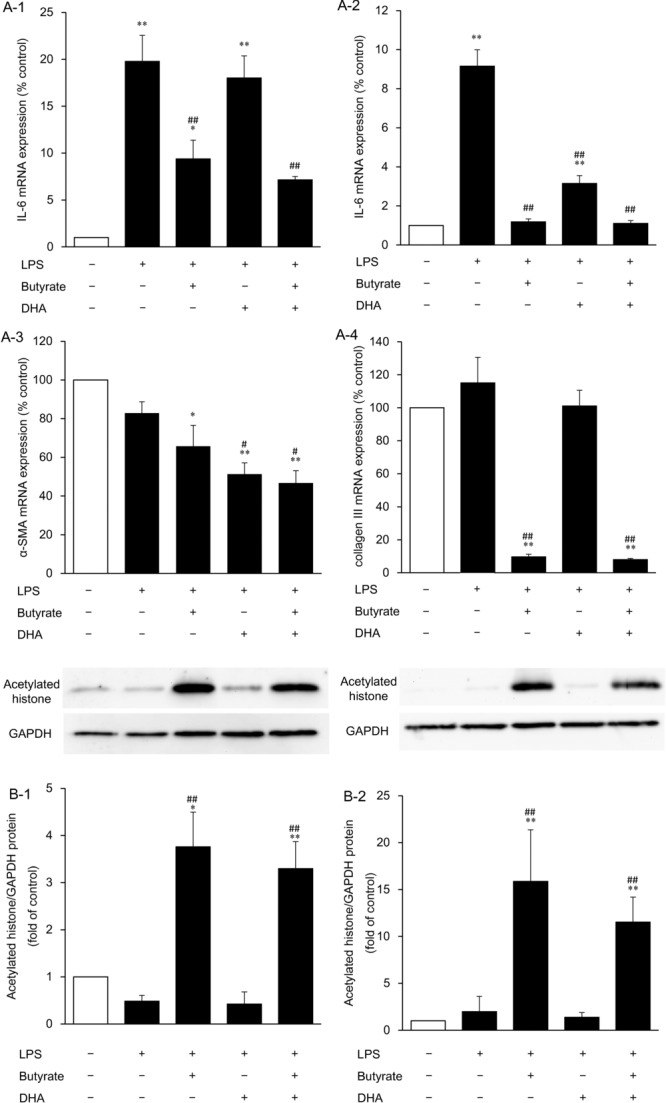
The effects of butyrate, DHA, and combined administration of butyrate and DHA on IL-6 mRNA expression at 3 (*a*-1) and 48 hours (*a*-2), α-SMA (*a*-3), and collagen III (*a*-4) mRNA expression levels at 48 hours, and histone acetylation at 3 (*b*-1) and 48 hours (*b*-2) after addition of each fatty acid to the culture medium. HDFs were exposed to DHA (100 μM), butyrate (16 mM), or butyrate with DHA (16 mM and 100 μM, respectively) for 3 or 48 hours after LPS addition to the culture medium. Data from 3 independent experiments were used to calculate mean values and SEMs. *P < .05, **P < .01 as compared with control cultures; *^#^P* < .05, *^##^P* < .01 in comparison with LPS alone (Tukey-Kramer post hoc test) (*a*). Acetylated histone protein was analyzed by Western blotting. A representative Western blot is shown at the top of each bar for each group and for the internal control. The graph presents the acetylated histone/GAPDH ratio. Data from 4 independent experiments were used to calculate mean values and SEMs. * *P* < .05, **P < .01 as compared with control cultures; *^##^P* < .01 in comparison with LPS alone (Tukey-Kramer post hoc test) (*b*). DHA indicates docosahexaenoic acid; α-SMA, α-smooth muscle actin; HDF, human dermal fibroblast; and LPS, lipopolysaccharide.

To evaluate the anti-inflammatory effects and the relation between anti-inflammatory and antifibrogenic effects, we analyzed IL-6 mRNA expression, which is reported to be upregulated in hypertrophic scars, and α-SMA and collagen III expression levels, which showed major changes under the influence of butyrate and DHA administration in LPS-unstimulated HDFs. In preliminary assays, significant increases in IL-6 mRNA expression were observed at 3, 6, 12, 24, and 48 hours after LPS addition to the medium. Therefore, we evaluated the effects of 16 mM butyrate and 100 μM DHA on IL-6 expression at 3 and 48 hours. Although LPS increased IL-6 expression at 3 and 48 hours by 20.0- and 9.2-fold, respectively (*P* < .01), administration of butyrate alone and combined administration of butyrate and DHA attenuated this upregulation at 3 and 48 hours (*P* < .01) and DHA alone did so at 48 hours (*P* < .01; Figs 6*a*-1 and 6*a*-2). As for the profibrotic factors, 16 mM butyrate alone or combined administration of butyrate and 100 μM DHA inhibited α-SMA (to 66% and 47% of the control level, respectively; *P* < .05) and collagen III (to 10% and 8% that of control level, respectively; *P* < .01) expression levels; DHA inhibited α-SMA expression (to 51% of the control level; *P* < .01) at 48 hours in the LPS-stimulated HDFs compared with the control level, whereas LPS did not increase α-SMA and collagen III expression in HDFs (Figs 6*a*-3 and 6*a*-4). Finally, we analyzed histone acetylation at 3 and 48 hours after fatty acid administration to evaluate butyrate's action as an HDAC inhibitor in LPS-stimulated HDFs. Administration of butyrate alone and combined administration of butyrate and DHA strongly increased histone acetylation not only at 48 hours (*P* < .01; Fig 6*b*-2) but also at 3 hours (*P* < .05; Fig 6*b*-1).

## DISCUSSION

This study shows the inhibitory effects on the expressions of profibrotic factors and proinflammatory factors by butyrate and DHA in HDFs, both individually and after combined administration. Butyrate had stronger inhibitory effects than propionate on α-SMA and collagen III expression, along with a strong reduction in cell proliferation, stress fiber formation, and LPS-induced IL-6 expression. DHA also inhibited mRNA expression of α-SMA and collagen III and LPS-induced IL-6 mRNA expression. The combined administration of butyrate and DHA augmented the inhibitory effects of butyrate on α-SMA and collagen III mRNA expression, cell proliferation, and stress fiber formation while decreasing the expression of collagen I mRNA. These findings are suggestive of the efficacy of butyrate and DHA as a combined treatment of dermal fibrosis.

In the present study, both butyrate and propionate exerted the inhibitory effects on the profibrotic factor expressions in HDFs, indicating a therapeutic potential of SCFAs against the dermal fibrotic response. The predominance of butyrate in this effect is in agreement with the results of our previous report on NF-κB regulation in peripheral blood mononuclear cells,^9^ pointing to the potency of physiological action of butyrate. Although TGF-β1 has been shown to augment α-SMA expression,^3^ butyrate did not alter TGF-β1 expression here. In contrast, butyrate strongly inhibited stress fiber formation in HDFs. Because stress fiber formation upregulates α-SMA expression in fibroblasts,^22^ the inhibitory effects on profibrotic factor expressions of butyrate are likely to be mediated by stress fiber alteration rather than cytokine regulation. As for anti-inflammatory effects, inhibition of IL-6 mRNA expression by butyrate was observed at 3 hours, as was greater histone acetylation in LPS-stimulated HDFs. This result is consistent with the report of Grabiec et al,^23^ showing IL-6 mRNA downregulation at 4 hours after administration of trichostatin A, a potent HDAC inhibitor. Therefore, it seems that IL-6 inhibition in the present study is due to the HDAC inhibitory action. LPS stimulation increased IL-6 expression but did not increase expression of profibrotic factors in HDFs. On the contrary, butyrate decreased expression levels of profibrotic factors in the LPS-stimulated group compared with the no-stimulation control. This inhibitory effect on profibrotic factors of butyrate therefore is likely to be independent from IL-6 regulation in this in vitro experiment on monolayer culture. However, this inhibitory action on IL-6 expression may exert therapeutic effects on scar tissue because the high expression of IL-6 in hypertrophic scars has been reported.^5^

We revealed the inhibitory effects on not only proinflammatory factors but also profibrotic factors by DHA in HDFs. ω-3 PUFAs have been shown to have anti-inflammatory effects.^15^ Along with these known effects, inhibition of IL-6 expression was observed in the present study. However, the independence of the inhibitory effects on profibrotic factors from that on proinflammatory factors was demonstrated in DHA-treated HDFs and butyrate-treated HDFs. Therefore, it appears that the inhibitory effects on profibrotic factor expressions of DHA are mediated by the mechanisms other than inflammatory factors. After the administration of DHA alone, TGF-β1 mRNA expression was significantly inhibited, as was α-SMA and collagen III mRNA expression. However, the dose dependence of the action of DHA on the α-SMA and collagen III mRNA expression levels was not consistent with that of TGF-β1. Therefore, regulation of TGF-β1 expression does not seem to be the primary mechanism underlying these inhibitory effects. In the present study, the antifibrogenic effect was exerted by DHA rather than EPA. D-series resolvins, protectins, and maresins are lipid mediators derived from DHA but not from EPA.^16^ Although the roles of these mediators in the DHA-induced inhibitory effects on profibrotic factors can be hypothesized, the direct inhibitory effects of these mediators on fibrogenic responses have not been demonstrated. To elucidate the mechanisms of DHA-induced inhibitory effects on profibrotic factors, detailed experiments examining the metabolism of ω-3 PUFAs and lipid mediators are required.

The cooperative inhibitory effect of combined butyrate and DHA administration on profibrotic factor expressions was revealed in the present study. DHA administration not only enhanced the antifibrogenic effects of butyrate but also inhibited collagen I mRNA expression by approximately 50%, indicating a more potent antifibrogenic effects than single fatty acid administration. Recently, the proapoptotic effect of combined butyrate and DHA administration on colonocytes was demonstrated.^24^ In the present study, proapoptotic morphological changes, such as DNA fragmentation or condensation, were not observed in the fatty acid–treated fibroblasts. Therefore, further studies are necessary to reveal this effect or its absence by means of primary culture of fibroblasts derived from pathological tissue such as keloids.

In the present study, we revealed the inhibitory effects on profibrotic factor expressions using the cultured human fibroblasts. Meanwhile, the mechanism of scaring in human is very complicated.^1^ Therefore, we cannot determine the antifibrogenic effects of these fatty acids in the present study. To develop the antifibrogenic therapy using these fatty acids, future clinical studies are required.

In summary, we report the inhibitory effects on profibrotic and proinflammatory factor expression by butyrate and DHA on HDFs, both individually and in combination. These findings can contribute to the development of novel therapies for dermal fibrosis. Further experiments are necessary to elucidate the mechanism behind these effects.

## Figures and Tables

**Table 1:** Primers used for real-time polymerase chain reaction

			Annealing,
Gene	Forward (5′-3′)	Reverse (5′-3′)	°C
GAPDH	CATCAAGAAGGTGGTGAAGC	CCTCCCCAGCAAGAATGTCT	62.5
α-SMA	CGTGGGTGACGAAGCACAG	GGTGGGATGCTCTTCAGGG	62.5
TGF-β1	GGGACTATCCACCTGCAAGA	CCTCCTTGGCGTAGTAGTCG	62.5
Collagen I	GTGCTAAAGGTGCCAATGGT	ACCAGGTTCACCGCTGTTAC	57.5
Collagen III	TATCGAACACGCAAGGCTGTGAGA	GGCCAACGTCCACACCAAATTCTT	65.8
IL-6	AAGCCAGAGCTGTGCAGATGAGTA	TGTCCTGCAGCCACTGGTTC	60.0
